# Texture Matters: Linking Sensory Properties of Fruit Gum Candies to Consumer Acceptance

**DOI:** 10.1002/fsn3.72267

**Published:** 2026-08-24

**Authors:** Maria Izaber‐Ludwig, Zinaida Catruc, Jonathan Beauchamp, Jessica Freiherr

**Affiliations:** ^1^ Department of Sensory Analytics and Technologies Fraunhofer Institute for Process Engineering and Packaging IVV Freising Germany; ^2^ Department of Psychiatry and Psychotherapy Friedrich‐Alexander‐Universität Erlangen‐Nürnberg Erlangen Germany

**Keywords:** check‐all‐that‐apply (CATA), consumer sensory testing, gelatine, plant‐based, vegan

## Abstract

The increasing popularity of plant‐based diets and snacks has led to a growing variety of alternative foods, yet these often fail to meet sensory expectations, which is a decisive factor for food acceptance. The confectionery sector has recognized this dietary transition and developed tailored products to meet consumer demands. Despite the variety of plant‐based confectioneries on the market, few studies have investigated these novel formulations for their sensory performance compared to their traditional counterparts, or how their sensory properties impact consumer acceptance. In this study, we selected fruit gum candies as a confectionery model to explore sensory perception and the modality‐specific liking in relation to overall acceptance. In addition to taste, our consumer study (*n* = 147), comprising 12 commercial products including various formulations, revealed that texture is an equally important driver of overall liking for fruit gum candies. Chewy gelatine‐based candies received the highest overall liking (mean ratings up to 7.3 on a nine‐point hedonic scale). Higher stickiness in starch‐ or pectin‐based candies decreased liking, while a harder texture improved the acceptance of products that used plant‐based gelling agents. The acceptance of gelatine decreased with increasing hardness. Acceptance of starch‐ and pectin‐based products decreased with consumer age by about 0.3–0.4 points per 10‐year increase in age, while gelatine‐based products maintained popularity. The present results highlight that texture, particularly hardness and stickiness, is an important driver of liking for fruit gum candies and provide a basis for optimizing plant‐based alternatives to meet sensory expectations.

## Graphical abstract

Sensory characterization and consumer acceptance testing (*n* = 147) of gelatine‐ and plant‐based fruit gum candies revealed that texture, alongside taste, is a key driver of overall liking. Gelatine‐based candies achieved the highest acceptance, while plant‐based alternatives were particularly favored by younger consumers. Created in BioRender. Izaber‐Ludwig, M. (2026) https://BioRender.com/bgo6n0f.

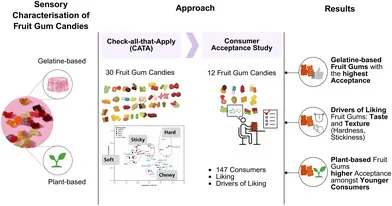

## 1 Introduction

Fruit gum candies represent a popular category of confectionery products appreciated for their variety in appearance, flavor, and texture, and are usually consumed to satisfy cravings for something sweet and to provide pleasure (Godshall 2015; Konar et al. 2022). The global sugar confectionery market amounted to approximately 123 billion USD in 2025 (Statista 2026), with gummy and jelly candies accounting for a major proportion and experiencing growing popularity (Vojvodić Cebin et al. 2024). In Germany, fruit gum candies and jelly products constitute an economically relevant segment of sugar confectionery production. The production value of fruit gum candies and jelly products manufactured in Germany reached 1.12 billion EUR in 2025, corresponding to a production volume of approximately 390,400 t (Federal Statistical Office 2025). The per‐capita consumption of sugar confectionery in Germany is reported to be 4.6 kg (Statista 2025).

Conventional fruit gum candies are often high in sugar content and offer limited nutritional value. The link between excessive sugar intake and the rising prevalence of overweight and obesity (Blüher 2025; World Health Organization 2022) has led to intensified efforts to reformulate confectionery products (Konar et al. 2022; Tarahi et al. 2023). Current reformulation strategies focus on sugar reduction and the incorporation of functional ingredients, like vitamins, probiotics, or plant extracts (Delgado and Bañón 2018; Mutlu et al. 2018; Rivero et al. 2020; Teixeira‐Lemos et al. 2021). In addition to these health‐oriented developments, there is a growing trend toward plant‐based alternatives, driven by plant‐based diets, rejection of animal‐derived ingredients for religious or ethical reasons, or food allergies (Konar et al. 2022). These reformulations often require substantial modifications to core ingredients, with direct consequences for the sensory properties of fruit gum candies.

A major challenge in developing reformulated products is their limited acceptance due to unfamiliar sensory properties in comparison to conventional products, whereby consumer‐specific factors also play a role (Giacalone et al. 2022; Gunes et al. 2022). In fruit gum candies, sensory properties are primarily determined by gelling agents that form the gel network (Burey et al. 2009). The resulting texture is an essential property for the acceptance of this product category (Kälviäinen et al. 2000). Gelatine has traditionally been the primary gelling agent, providing a clear, elastic and thermally reversible gel whose textural properties are highly accepted and enjoyed by consumers (Imeson 2011; Mohos 2010; Wang and Hartel 2022). Plant‐based fruit gum candies, formulated with gelling agents like starch or pectin, have evolved in Western markets over the last 15 years (Konar et al. 2022). Achieving an appropriate texture remains challenging, as plant‐based gelling agents often produce stickier or softer textures with altered elasticity (Burey et al. 2009; Marfil et al. 2012). Well‐formulated plant‐based systems can closely mimic gelatine‐like properties, however, and thereby achieve high consumer acceptance (de Avelar and Efraim 2020; Rawat et al. 2024). Texture can be further modified by other ingredients, such as sweeteners, fibers or adjusted water content, with direct consequences on appearance and flavor release (de Avelar and Efraim 2020; Boland 2004; Hartel et al. 2018; Pereira et al. 2022; Tarahi et al. 2023). Indeed, in addition to texture, key sensory dimensions for acceptance are a vibrant color, transparency and glossy surface, balanced sweetness and natural flavors (Cano‐Lamadrid et al. 2020; Gunes et al. 2022).

The use of alternative sweeteners has been studied in view of reducing or replacing the high sugar content of conventional fruit gum candies, with alternative sweeteners that influence sweetness intensity and quality (Maringka et al. 2024; McKenzie and Lee 2022; Rivero et al. 2023). Fruit purée or juice addition offers further ways to modify flavor and provide functional components (Figueroa et al. 2025; Renaldi et al. 2022; Teixeira‐Lemos et al. 2021). Since perception is multisensory, all sensory modalities contribute to overall acceptance, albeit to varying degrees and with differing importance, depending on the food. Taste and flavor are usually cited as the most important characteristics for acceptance (Andersen et al. 2019; Moskowitz and Krieger 1995). For fruit gum candies, flavor and texture are consistently cited as dominant factors for their acceptance (Gunes et al. 2022), but previous studies have not examined the relative importance of the individual sensory liking modalities to overall acceptance. Fruit gum candies provide a case study of the relationship between ingredients, sensory perception and consumer acceptance, as they have limited ingredients impacting sensory properties.

Consumer acceptance and openness toward reformulated foods are not only influenced by intrinsic product characteristics, but also by consumer‐related factors, such as education, sex, and age (Giacalone et al. 2022; Kälviäinen et al. 2000; Slade 2018). The degree of acceptance of a new formulation depends on previous experiences and established preferences, although acceptance is not static and can be enhanced by increasing familiarity (Schicker et al. 2023). Improving acceptance of reformulated foods may therefore contribute positively to a healthier food system.

Despite the emergence of novel plant‐based fruit gum candies, studies on consumer acceptance have hitherto focused mainly on single optimized formulations with specific ingredient combinations. Consequently, broader evaluations of consumer acceptance of fruit gum candies using real‐market samples are sparse. While many studies highlight individual sensory drivers of acceptance and demonstrate that novel products can achieve high liking scores (de Avelar and Efraim 2020; Nishiyama‐Hortense et al. 2022; Rivero et al. 2021, 2023), evaluations of whether these products meet consumer expectations or how they compare to benchmark products are lacking. In addition to texture, main drivers of liking are color and taste, but there is limited understanding of how the combination of these modalities relates to consumer acceptance. Previous studies rarely compared individual sensory inputs within a multisensory experience or the relationship between ingredients, sensory perception and consumer acceptance. In most cases, only individual aspects of these relationships were explored on account of small panel sizes, limited ranges of sensory properties or an absence of information on the consumers (Čižauskaitė et al. 2019; Mutlu et al. 2018; Tireki et al. 2021; Vojvodić Cebin et al. 2024). The novelty of this study lies in its broader perspective, examining a diverse range of fruit gum candies and combining different methods to evaluate their sensory properties and consumer acceptance. It contributes to the literature by investigating how the liking of individual sensory modalities relates to overall product acceptance.

The present study aimed to investigate sensory perception and consumer acceptance of multiple selected fruit gum candies available in the German market. The main objectives were to explore which sensory properties are the main drivers of acceptance and to investigate the relationship between liking of individual sensory attributes and overall product liking. Furthermore, this study sought to provide insights into potential differences in sensory acceptance across various consumer groups. These objectives were explored using a wide range of commercially available fruit gum candies, including both conventional and plant‐based alternative products. The fruit gum candies were first evaluated by a trained sensory panel to characterize the products' sensory space and highlight key sensory qualities. The resulting sensory map was used to select 12 representative samples for subsequent evaluations of product acceptance by a larger, naïve consumer panel. The consumer study established a baseline for acceptance of selected fruit gum candies with conventional ingredients to offer potential areas for improvement in the sensory properties of novel products.

## 2 Material and Methods

### 2.1 Samples

Thirty representative commercial products from the German soft confectionery market were chosen for initial evaluation (P1—P30; Table 1). The sample set was compiled from a shelf survey of major supermarket retailers in southern Germany and covered leading national brands as well as private‐label products across different price segments. The samples represent a wide range of typical fruit gum candies and were selected based on the following criteria: gelling agent (gelatine, *n* = 13; starch, *n* = 7; pectin, *n* = 6; blends, *n* = 3; gum arabic, *n* = 1), sweetener (e.g., sugar, reduced sugar, polyols) and further ingredients (e.g., fibers or fruit juice content). Gum arabic was present in only one commercial product (P27) in the market survey. This product was retained in the sample set because it represents a commercially available plant‐based fruit gum candy with a distinctly hard texture. Due to its single representation (*n* = 1), however, gum arabic was not treated as a separate gelling‐agent category in the consumer evaluation. Although the sample set captures the major commercial categories present on the German market at the time of the survey, it does not aim to be a comprehensive set of all available products. Information on the ingredients, in particular the origin and type of gelling agents, was collected from information on the product labels. The gelling agent was labeled as porcine gelatine in four samples (Table 1), with the remaining products not disclosing this information.

**TABLE 1:** Fruit gum candies with main ingredients.

Texture cluster	ID	Gelling agent	Sweetener^a^	Sugar content (%w/w)^3^	Fruit juice content^b^	Coating
SO	P3	Pectin	Sugar	62	No	Carnauba wax
	P14	Starch	Sugar	62	Yes (20% fruit juice)	Blend (beeswax, carnauba wax)
	**P18**	**Starch**	**Sugar**	**53**	**Yes (5% fruit juice, 5% vegetable juice)**	**Blend (beeswax, carnauba wax)**
	P20	Pectin	Sugar	58	No	Carnauba wax
	**P21**	**Pectin**	**Sugar**	**57**	**Yes (1.7% fruit juice)**	**Carnauba wax**
	P22	Pectin	Sugar	56	Yes (21% fruit juice)	Carnauba wax
	P25	Pectin	Sugar	55	No	Carnauba wax
	**P30**	**Pectin**	**Sugar**	**59**	**No**	**Carnauba wax**
ST	P2	Starch	Polyol	< 0.5	No	Carnauba wax
	P8	Starch	Sugar	42	No	Beeswax
	P11	Starch	Sugar	58	No	Carnauba wax
	**P12**	**Starch**	**Reduced sugar**	**31**	**No**	**Beeswax**
	**P13**	**Starch**	**Sugar**	**59**	**No**	**Carnauba wax**
	P15	Blend (starch, pectin)	Sugar	60	No	Carnauba wax
	P16	Blend (starch, pectin)	Sugar	60	No	Carnauba wax
	**P24**	**Blend (gelatine, starch)**	**Sugar**	**56**	**No**	**Beeswax**
CH	**P4**	**Gelatine**	**Sugar**	**58**	**Yes (7% fruit juice)**	**Carnauba wax**
	P9	Gelatine	Sugar	46	No	Beeswax
	P10	Gelatine	Sugar	46	Yes (25% fruit juice)	Beeswax
	P17	Gelatine (porcine)	Reduced sugar	31	No	Blend (beeswax, carnauba wax)
	**P19**	**Gelatine**	**Sugar**	**52**	**Yes (5% fruit juice)**	**Carnauba wax**
	P23	Gelatine (porcine)	Sugar	56	Yes (20% fruit juice)	Carnauba wax
	**P28**	**Gelatine** (porcine)	**Sugar**	**48**	**No**	**Blend (beeswax, carnauba wax)**
	P29	Gelatine	Sugar	43	No	Blend (beeswax, carnauba wax)
HA	**P1**	**Gelatine**	**Polyol**	**< 0.5**	**Yes (2% fruit juice)**	**Blend (beeswax, carnauba wax)**
	**P5**	**Gelatine**	**Sugar**	**39**	**Yes (20% fruit juice)**	**Carnauba wax**
	P6	Gelatine	Polyol	< 0.5	No	Carnauba wax
	**P7**	**Gelatine**	**Sugar**	**46**	**No**	**Carnauba wax**
	P26	Gelatine (porcine)	Sugar	48	Yes (20% fruit juice)	Carnauba wax
	P27	Gum arabic	Sugar	40	Yes (30% fruit juice)	Carnauba wax

*Note:* Samples in bold were chosen for the consumer acceptance study.

Abbreviations: CH, chewy texture; HA, hard texture; SO, soft texture; ST, sticky texture.

^a^ Coding of sweetener simplified (e.g., sugar corresponds to glucose‐fructose syrup, glucose syrup, or sucrose).

^b^ Sugar and fruit juice contents were obtained from the sample declaration.

Samples were purchased with similar best‐before dates. They were stored in their original packaging at room temperature and were freshly opened directly before each sensory evaluation. All samples were blinded using a random, unique three‐digit code in both evaluations. Presentation of the fruit gum candies to the sensory panelists varied according to the evaluation: for the trained panel, two candies of each sample were served loosely on a tray, whereas for the consumer study, samples were presented in closed glass jars. The amount and number of samples evaluated per sitting were limited to prevent sensory fatigue of panelists, as well as to minimize sugar intake.

Sensory evaluation by the trained panel was used to categorize all 30 samples according to their predominant texture attributes (Table 1) and to reduce the sample set for the consumer study to 12 representative samples. These were selected to cover the sensory diversity of the full sample set while keeping the consumer test feasible (detailed description of sample choice in Section 3.2.1).

### 2.2 Sensory Evaluations

The study was approved by the local ethics committee of the Friedrich‐Alexander‐Universität Erlangen‐Nürnberg (reference no. 23‐331‐S, 19/9/2023) and conformed with the Declaration of Helsinki. Participation in the study was voluntary and all participants—both trained and naïve panelists—provided their prior written informed consent.

### 2.3 Check‐All‐That‐Apply Evaluation

The focus of the trained panel evaluations was to provide a rapid screening of a large variety of samples, not on a full sensory descriptive analysis. For this purpose, the check‐all‐that‐apply (CATA) method was used for assessing 30 samples, from which a subset of samples was selected for the subsequent consumer study.

Ten panelists (7 females, 3 males; mean age 31 ± 9 years, aged 23 to 55 years) from the Fraunhofer IVV sensory panel evaluated all 30 fruit gum candy samples in one session without replication. Panelists self‐reported having normal olfactory function and no known illness at the time of the sensory tests. Normal olfactory function was confirmed by the panelists' performance in routine sensory training. Panelists were trained in the descriptive evaluation of aroma and taste of food products according to DIN EN ISO 8586 (Deutsches Institut für Normung 2023) and had previous experience in the sensory evaluation of confectionery, including the CATA methodology. Although CATA is preferentially used with naïve consumers and a high number of participants (Ares et al. 2014), previous work has shown that CATA and related rapid descriptive methods can provide robust and reproducible sample descriptions, even when used with semi‐trained or smaller panels (Alexi et al. 2018; Giacalone and Hedelund 2016). In this study, the CATA evaluation with 10 trained panelists was used as a rapid screening method to map the sensory space of 30 products and to guide sample selection for the consumer study, rather than to provide fully generalisable descriptive data for the wider population. The panelists completed a CATA questionnaire that listed 14 attributes for all 30 samples. Attributes primarily focused on texture and included terms within the modalities of appearance, taste and flavor (Table 2). These were preselected by a focus group of three trained panel members, each with extensive expertise in the sensory evaluation of food. Although typically between 10 and 30 attributes are used in CATA studies (Jaeger et al. 2015), the list here was restricted to 14 attributes to keep the task manageable for the panelists. Moreover, qualitatively different texture categories were used for attributes like “smooth/medium/tough bite” and “soft/medium/hard while”. This choice is a trade‐off between discriminative power and a simpler task that is more appropriate for screening large quantities of samples within one session. During the session, panelists tasted the 30 samples individually and were asked to check all applicable items from the shortlist of attributes. Sample presentation was randomized for each panelist using a Williams design to control for order effects. Participants had to take breaks between samples to refresh their palate using still water and unsalted crackers.

**TABLE 2:** Selected 14 sensory attributes for initial sample characterization via the check‐all‐that‐apply procedure.

Sensory modality	Appearance	Flavor	Taste	Texture	Texture
				First bite	Chewing
Attribute	Glossy Matt Neither matt nor glossy	Fruity	Sweet Sour	Smooth bite Medium bite Tough bite	Soft Medium hard Hard Sticky Chewy

### 2.4 Consumer Acceptance

Consumers (*n* = 147; 85 females, 62 males; mean age 34 ± 13 years; age range 18 to 67 years) were recruited during the “Long Night of Sciences” (Lange Nacht der Wissenschaften) public outreach event that took place at the Friedrich‐Alexander‐Universität Erlangen‐Nürnberg on 21st October 2023. A screening questionnaire for self‐reported eligibility ensured the following inclusion criteria for the study: age between 18 and 70 years; no neurological or psychological conditions; no food allergies or intolerances; no diabetes; or no conditions affecting taste, smell or flavor perception and metabolism. Furthermore, participants were excluded if they were pregnant, breastfeeding or unwilling to consume animal‐derived products. Dietary preferences (including vegetarianism/veganism) were recorded; participants following such diets provided explicit consent to taste gelatine‐based samples. Participants were informed of the nature of the study (consumer acceptance of a variety of fruit gum candies) and gave written consent to participate. Participants who met all inclusion criteria answered the sample questionnaire anonymously using their personal smartphones. Data were collected via LimeSurvey (LimeSurvey GmbH, Germany).

For the consumer acceptance test, 12 samples were selected from the initial product set to represent the sensory diversity, as identified by the trained panel. This number was considered a trade‐off between broad product range and the feasibility of the consumer study. Furthermore, participants did not evaluate all 12 samples. Instead, an incomplete design was chosen to prevent sensory fatigue and to keep the evaluation short. Each participant evaluated a subset of four of the 12 samples (4‐of‐12 per participant). Samples were selected based on differences in CATA profiles and representativeness of formulation types. Randomization was stratified by texture cluster (one randomly chosen out of three samples per cluster: soft texture (SO), sticky texture (ST), chewy texture (CH), hard texture (HA)), ensuring texture diversity but potentially inducing unequal per‐product evaluations and cluster‐wise balancing. The samples were presented monadically with randomization at participant level. Due to the incomplete design, each of the 12 samples was evaluated by 34–62 consumers (mean *n*≈49; see Table S2). As the samples were always mixtures of different colors and flavors, the study instructors randomly selected one fruit gum candy from the jar corresponding to the number indicated in the questionnaire. Within‐pack variance in color and flavor was not controlled. Participants evaluated color, aroma (orthonasally), taste, fruitiness, hardness, and stickiness liking, and rated their overall liking of the samples on a nine‐point hedonic scale (1 = “dislike extremely”, 5 = “neither like nor dislike”, 9 = “like extremely”) (Peryam and Pilgrim 1957). Five‐point just‐about‐right (JAR) scales were used to evaluate which adjustments the fruit gum candies needed to achieve higher acceptance. The JAR‐level of color, aroma, taste, sweetness, fruitiness, stickiness and hardness was scored (e.g., color: 1 = “too light”, 3 = “just about right”, 5 = “too dark”) (Varela and Ares 2014). Participants in the study were rewarded with a snack for their participation.

In addition to answering demographic questions, participants were prompted to provide information about their consumption frequency of sweets and fruit gum candies. Education level was not recorded as a consumer characteristic. 75.0% of the consumer panel were between 24 and 42 years old. 13.6% self‐reported as being on a vegetarian or vegan diet, which is in line with reported proportions of vegetarians (around 7%–10%) and vegans (1%–2%) in the German population (Gimpfl et al. 2025). About 69.4% of participants reported consuming sweets daily or two to three times a week (Table 3). Fruit gum candies were consumed less frequently, with the largest proportion (54.4%) reporting consumption once a month. These data indicate that the panel predominantly consisted of regular consumers of sweets but occasional consumers of fruit gum candies.

**TABLE 3:** Reported consumption frequency of sweets and fruit gum candies (*n =* 147).

	Consumption frequency of sweets	Consumption frequency of fruit gum candies
Never	1 (0.6%)	9 (6.1%)
Once a month	2 (1.4%)	80 (54.4%)
Once a week	42 (28.6%)	39 (26.5%)
2*–*3 times a week	50 (34.0%)	16 (10.9%)
Daily	52 (35.4%)	3 (2.1%)

### 2.5 Statistical Analyses

Cochran's *Q*‐test was employed to test for significant attributes in the CATA dataset from the initial characterization of the fruit gum candy samples (Cochran 1950). Correspondence analysis (CA) was conducted on the contingency table with the resulting significant attributes (see Figure S1) using Chi‐square distance to create a sensory map (Meyners et al. 2013). Samples were categorized using coordinates from CA via agglomerative hierarchical cluster (AHC) analysis using Euclidean distance and Ward's method. The number of clusters was identified based on the resulting dendrogram and the Hartigan index (see Figure S2 and Table S1).

The distances between the samples in the CA were used to select the samples for the consumer study. For the evaluation of the consumer data, mean values and corresponding standard deviations of the different liking ratings were calculated for each sample (see Table S2). Linear mixed models (LMMs) were chosen to reflect the unbalanced study design and account for the repeated assessments of participants (Lahne et al. 2014). LMMs were fitted per attribute to assess differences in the liking of the sensory attributes between samples (see Tables S3 and S4). The respective liking ratings were considered as the dependent variable and different samples were treated as fixed factors. Participants were treated as random factors to account for the evaluation of multiple samples. For all post hoc tests, the Holm method was applied to adjust *p*‐values for multiple comparisons of the samples and degrees of freedom were approximated using the Kenward–Roger method.

To investigate the contribution of the individual liking of sensory attributes on overall liking, a LMM was fitted with overall liking as dependent variable and the liking ratings of color, aroma, taste, fruitiness, hardness and stickiness as fixed effects. Products and participants were treated as random factors (see Table S5). Mean values and standard deviations of the JAR data were calculated for each texture cluster to further explore the drivers of liking (see Table S6). Ratings (1 = “too little”, 3 = “just about right”, 5 = “too much”) were centred at the JAR level (−2 = “too little”, 0 = “just about right”, 2 = “too much”) and were used together with the gelling agent as fixed effects in the LMM, with overall liking as the dependent variable. Samples and participants were random factors. Two‐way interactions of gelling agent with JAR ratings were considered and non‐significant effects were eliminated in a stepwise approach (Kuznetsova et al. 2017) (see Tables S7 and S8). A supportive penalty analysis was conducted on the overall liking rating and JAR responses. These were clustered according to the AHC analysis of the initial sensory properties (Pagès et al. 2014). A significant impact on overall liking was considered if more than 20% of consumers deviated from “just about right” in their rating and caused a mean drop in the rating of more than 1 point (Popper 2014).

To identify influences of sociodemographic factors on liking, a LMM was estimated with overall liking ratings as the dependent variable and gelling agent (gelatine, starch, pectin and blend) as fixed factors. Age (centred) and sex were used as additional fixed factors in the model to assess differences in consumer evaluation behavior related to demographics. Samples and participants were treated as random factors. Results for pectin (*n* = 2 products) and blend (*n* = 1) were treated as exploratory due to limited sample sizes (see Table S9).

Statistical analyses were conducted using XLSTAT (version 2022.3.2, Addinsoft, Paris, France) and R (version 4.2.2, R Core Team, 2022, Vienna, Austria). LMMs were modeled using the *lme4* (Bates et al. 2015), *lmerTest* (Kuznetsova et al. 2017) and *emmeans* (Lenth 2025) R‐packages (see Tables S3 and S4). The plots were created using OriginPro (Version 2025, OriginLab Corporation, Northampton, MA, USA), *ComplexHeatmap* (Gu et al. 2016) and *ggplot2* (Wickham 2016) R‐packages. *p‐*Values of 0.05 and lower were considered significant.

## 3 Results

### 3.1 Sensory Characterization of Full Sample Set

**FIGURE 1:**
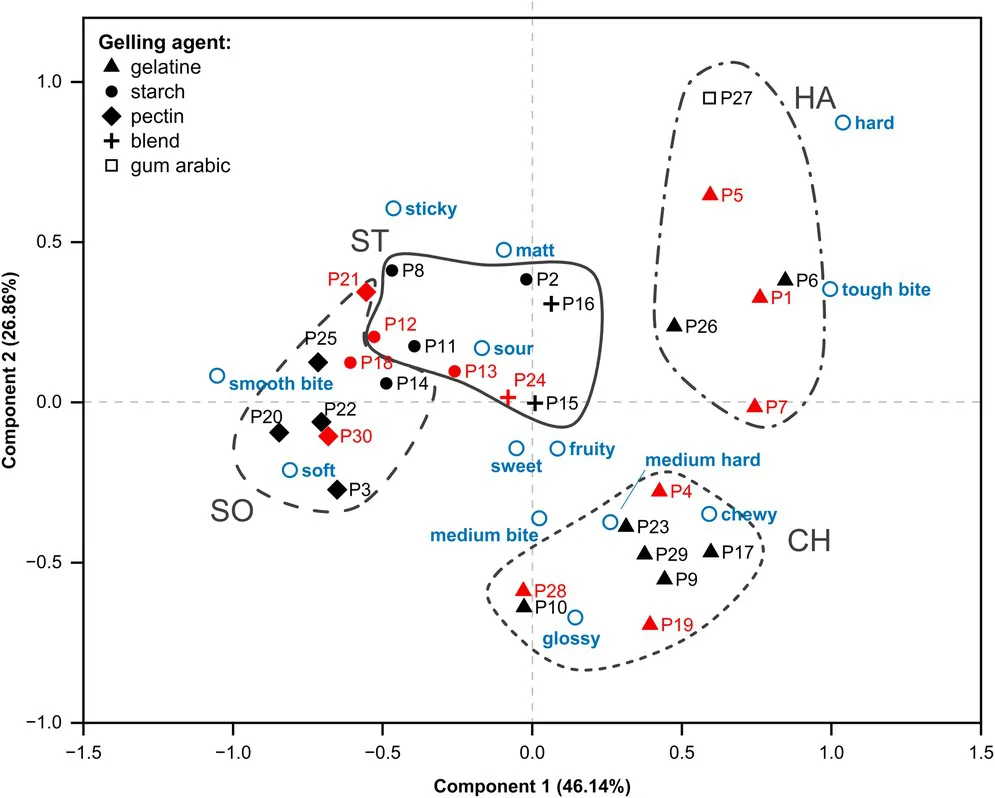
Correspondence analysis biplot of the check‐all‐that‐apply evaluation by the trained sensory panel, illustrating the sensory space of the 30 fruit gum candy products (P1‐P30). Significant sensory attributes (*n* = 13, *p* < 0.05) are highlighted (blue circles). Clusters from agglomerative hierarchical clustering are named by texture during chewing (Component 1) and indicated with different lines, SO, soft texture (dashed); ST, sticky texture (solid); CH, chewy texture (dotted); HA, hard texture (dash‐dotted). Samples depicted in red were chosen for the consumer study.

The 30 fruit gum candy samples were evaluated by CATA according to 14 attributes. The most frequently chosen term was the flavor attribute “fruity” (204 times) while the least‐used term related to appearance with the attribute “neither glossy nor matt” (23 times). Correspondence analysis was performed with 13 attributes. The term “neither glossy nor matt” was excluded, as it was the only non‐significant attribute (Cochran's *Q*‐test). Overall, 73.0% of the total variance in the dataset was explained by the first two dimensions (Figure 1). The first dimension (Component 1) was primarily associated with texture perception at the first bite, particularly toughness. Samples with harder textures were positioned on the positive side of the axis, while softer fruit gum candies were located on the negative side. The second dimension (Component 2) also contributed to the differentiation of the samples, although it was not dominated by a single sensory modality. This dimension was mainly associated with appearance and taste attributes, with appearance providing greater discrimination than taste. Texture‐related attributes perceived during chewing, such as stickiness, chewiness, medium hardness, and hardness, were separated along Component 2. Overall, Component 2 reflects a combination of appearance, taste and secondary texture‐related differences among the samples.

Cluster analysis revealed four clusters that were primarily described by texture attributes (Component 1) and broadly align with the different types of gelling agents used in fruit gum candies. The other ingredients did not support this grouping, nor did the coating correspond to the appearance distribution. The clusters were therefore named according to their texture attribute while chewing: “Soft” (SO), “Sticky” (ST), “Chewy” (CH), “Hard” (HA).

The first cluster, SO, was defined by a “smooth bite” and “soft” texture while chewing. Some of these samples were assessed as “sticky”, but this attribute was not unique to this cluster. The SO cluster consisted of samples with pectin and starch as a gelling agent. The ST cluster consisted mostly of starch‐based fruit gum candies or starch blends (starch/pectin: P15, P16; gelatine/starch: P24). These samples exhibited no “tough bite” or “chewy” texture but were evaluated as being “sticky” in texture and “matt” in appearance. Samples exhibiting “medium bite” and “medium hard” texture while chewing, as well as a “chewy” texture, form the CH cluster that was made up of only gelatine‐based fruit gum candies. The final HA cluster was characterized by the texture attributes “tough bite” and “hard” and included samples with gelatine as a gelling agent. One exception in this cluster is sample P27, which used gum arabic as a gelling agent and was not described as “chewy” but as the hardest in the sample set.

### 3.2 Consumer Acceptance

#### 3.2.1 Sample Selection

A selection of representative samples to cover a diverse range of fruit gum candies was chosen from the initial sample set for the consumer acceptance evaluations. Samples were selected to include the most discriminating sensory attributes identified in the sensory map and its ensuing clusters. Each cluster was assumed to be sufficiently homogeneous to represent specific properties of fruit gum candies, with the hypothesis that samples within a cluster would receive comparable liking ratings. Selections were made based on the number of mentions in the contingency table and choosing samples that differed from each other to cover a wide range of properties from each cluster. Additionally, the availability of samples at the time of the study was considered. This led to the selection of samples highlighted in bold in Table 1.

#### 3.2.2 Consumer Acceptance of Fruit Gum Candies

**FIGURE 2:**
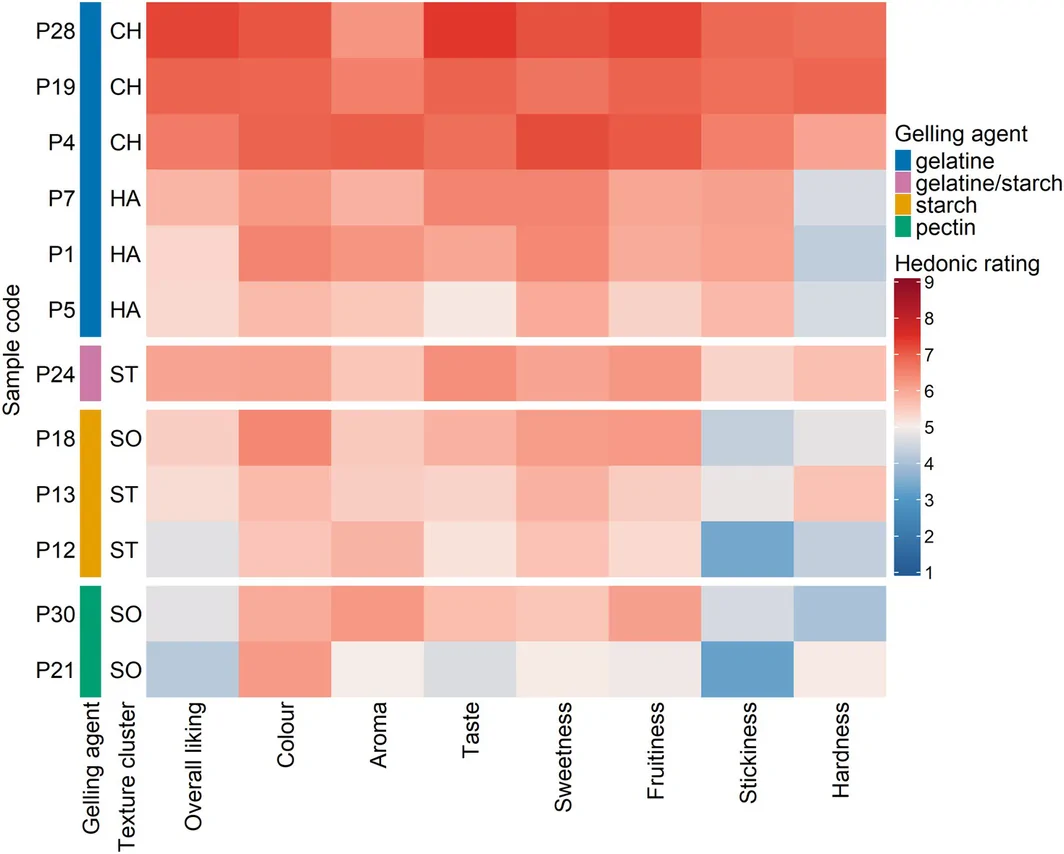
Heatmap of mean hedonic ratings on a nine‐point hedonic scale for overall liking and attributes across 12 selected fruit gum candy products (P). Colors range from blue (1) to white (5) to red (9); samples are ordered according to their gelling agent (gelatine, *n* = 6; blend (gelatine/starch), *n* = 1; starch, *n* = 3; pectin, *n* = 2). Clusters from agglomerative hierarchical clustering are indicated: SO, soft texture; ST, sticky texture; CH, chewy texture; HA, hard texture. See Table S2 for *n*‐per‐sample, standard deviations, and post hoc group letters.

Mean ratings of the overall liking for the representative 12 fruit gum candies ranged between 4.17 and 7.29 and differed significantly from each other (F_11,527_ = 11.63, *p* < 0.001). The least liked sample was the pectin‐based sample P21 while the most liked was the gelatine‐based sample P28 (Figure 2 and Table S2). Among the 12 samples, eight had mean ratings between 5 and 7 (“neither like nor dislike” and “like moderately”, respectively) and were made of different gelling agents, like gelatine (P4) or starch (P13). Liking of the individual attributes between samples largely mirrored overall liking (liking of color (F_11,535_ = 3.67, *p* < 0.001), aroma (F_11,535_ = 3.94, *p* < 0.001), taste (F_11,512_ = 9.10, *p* < 0.001), sweetness (F_11,520_ = 5.56, *p* < 0.001), fruitiness (F_11,528_ = 6.75, *p* < 0.001), stickiness (F_11,550_ = 16.22, *p* < 0.001) and hardness (F_11,552_ = 8.93, *p* < 0.001)). The most‐liked sample (P28, gelatine) also ranked high for taste, fruitiness and hardness. In contrast, the least‐liked sample (P21, pectin) was rated lower for taste, fruitiness and stickiness. Within the gelatine‐based fruit gum candies, samples differing in hardness/chewiness (HA vs. CH clusters) showed corresponding differences in hardness liking and overall liking.

### 3.3 Drivers of Overall Liking

#### 3.3.1 Influence of Attribute Liking on Overall Liking

The LMM exploring the contribution of the different liking ratings of sensory attributes (color, aroma, taste, sweetness, fruitiness, stickiness, hardness) on overall liking showed that liking of taste (*β* = 0.36, F_1,577_ = 112.76, *p* < 0.001) and hardness (*β* = 0.24, F_1,383_ = 111.13, *p* < 0.001) had the strongest positive associations with overall liking. Liking of fruitiness and stickiness also showed significant positive effects (*β* = 0.17 and 0.15 per scale point, respectively; F_1,577_ = 28.65, *p* < 0.001 and F_1,284_ = 34.82, *p* < 0.001), followed by sweetness liking (*β* = 0.10, F_1,579_ = 9.01, *p* = 0.003). Color liking had a smaller but significant effect (*β* = 0.06, F_1,577_ = 5.29, *p* = 0.022), whereas aroma liking did not contribute significantly to overall liking (F_1,571_ = 0.36, *p* = 0.55). Overall, these results indicate that flavor‐ and texture‐related liking, particularly taste, hardness, stickiness, and fruitiness, are the main drivers of overall acceptance in this product category.

#### 3.3.2 Exploratory Just‐About‐Right Analysis by Gelling Agent and Texture Perception

Mean ratings and standard deviations for the JAR questions were calculated for the four clusters of samples (see Table S6). To investigate how sensory attributes contribute to the overall acceptance, JAR‐level (color, aroma, taste, sweetness, fruitiness, stickiness, and hardness) and ingredients (gelling agents) were used as fixed effects to create an LMM. The results are exploratory due to the limited sample size per gelling agent.

**FIGURE 3:**
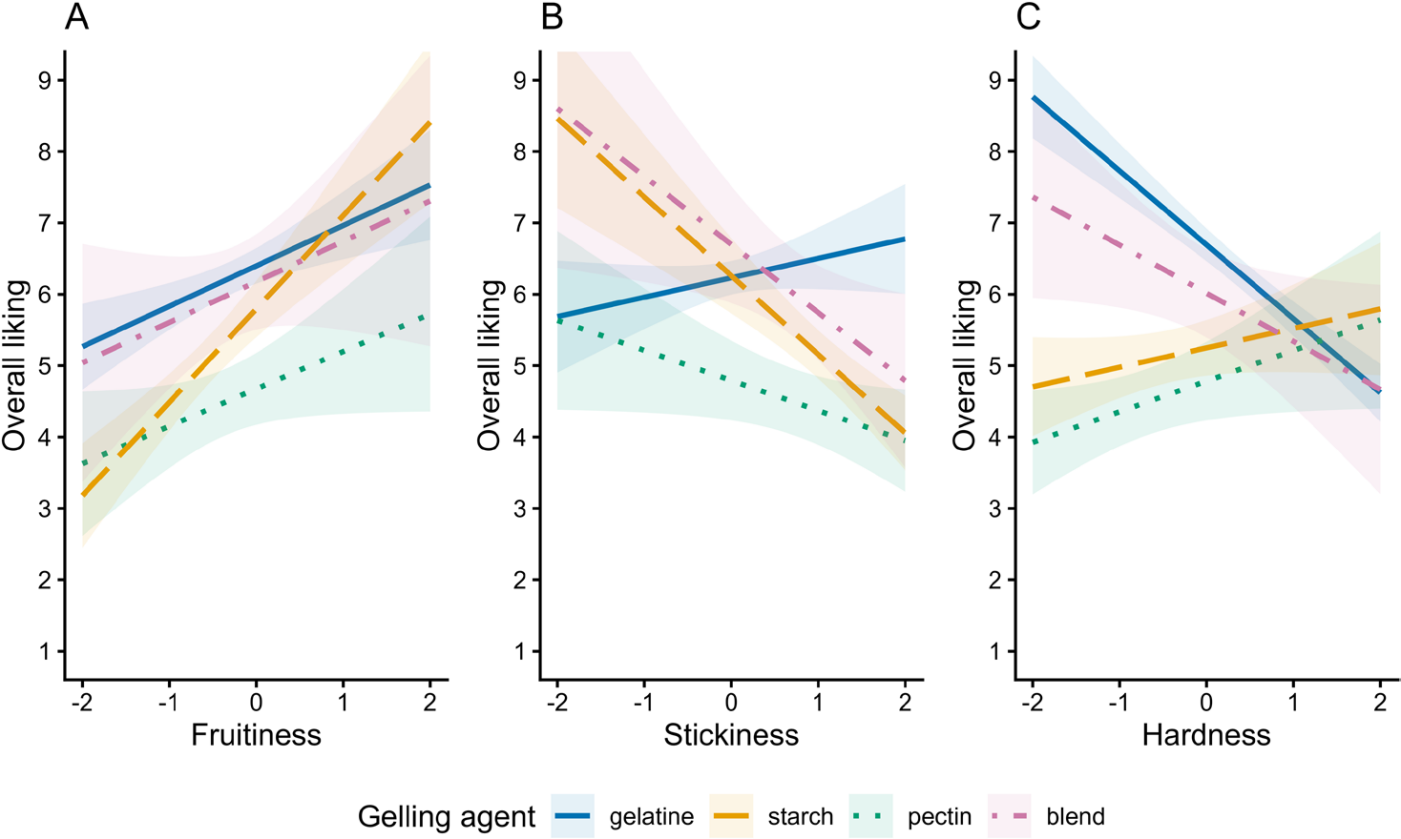
Associations between just‐about‐right (JAR) attributes and overall liking (nine‐point hedonic scale): Fruitiness (A), Stickiness (B), and Hardness (C). Lines are linear regressions with 95% confidence bands; JAR ratings are centred around just‐about‐right (−2 too little, 0 just‐about‐right, +2 too much). Colors and line types denote gelling agents (gelatine, starch, pectin, blend). Estimates are exploratory due to limited sample size per gelling agent.

Significant interactions were observed for gelling agent with fruitiness (F_3,525_ = 3.73, *p* = 0.011), gelling agent with stickiness (F_3,542_ = 5.97, *p* < 0.001) and gelling agent with hardness (F_3,440_ = 19.88, *p* < 0.001) (Figure 3 and Table S8). Increasing JAR‐level of fruitiness was associated with higher liking for all gelling agents, with the largest effect for starch samples (*β* = 1.30 per JAR‐level, *p* < 0.001, 95% CI [0.89, 1.71]). Increased JAR‐level for stickiness reduced liking for starch (*β* = −0.89 per JAR‐level, *p* < 0.001, 95% CI [−1.25, −0.52]) and showed a weak negative trend for pectin (*β* = −0.37 per JAR‐level, *p* = 0.041, 95% CI [−0.73, −0.01]), while showing only little effect on gelatine. Reduced JAR‐level for hardness decreased overall liking for gelatine (*β* = −0.91 per JAR‐level, *p* < 0.001, 95% CI [−1.17, −0.65]) and increased liking for starch and pectin.

Significant main effects were observed for aroma (F_1,565_ = 7.90, *p* = 0.005) and taste (F_1,569_ = 9.24, *p* = 0.002). Moving above JAR in aroma slightly increased overall liking, whereas moving below JAR in taste was associated with higher overall liking across gelling agents.

**FIGURE 4:**
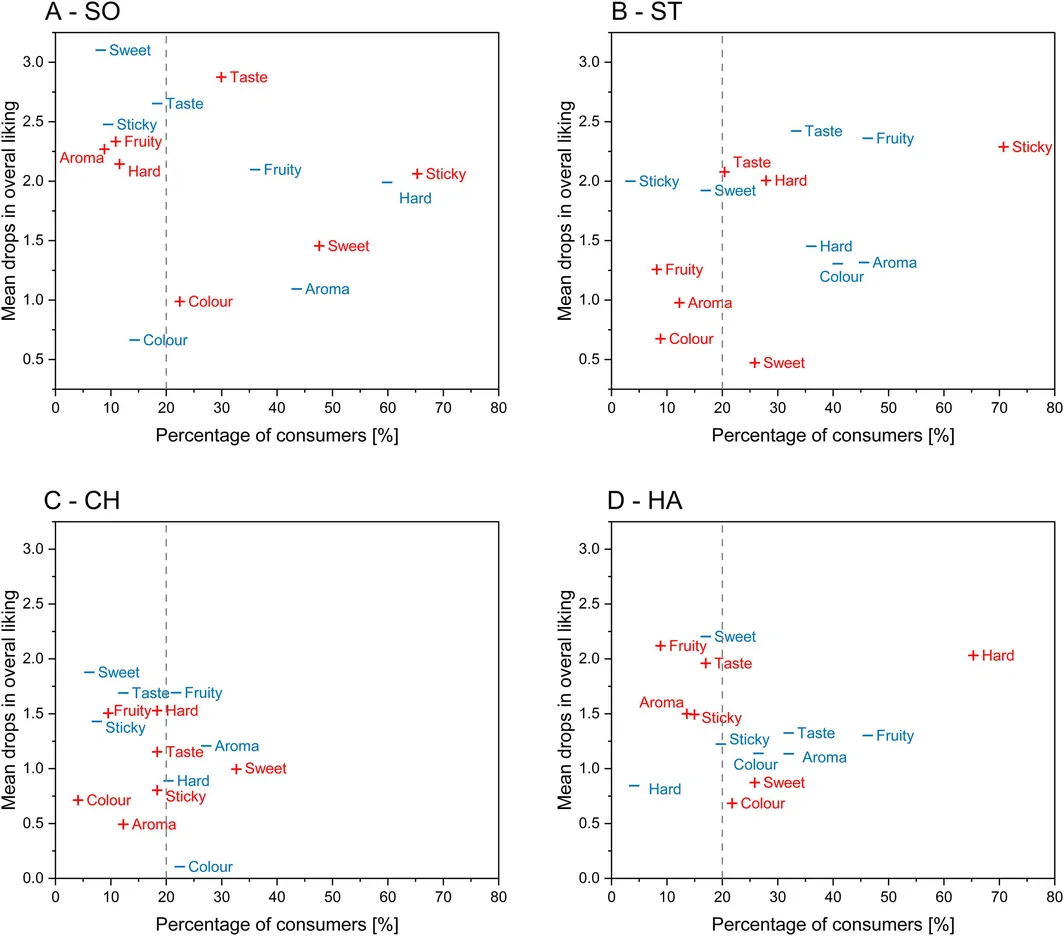
Penalty analysis data with attributes and their influence on overall liking (A) SO, soft texture; (B) ST, sticky texture; (C) CH, chewy texture; (D) HA, hard texture; dashed vertical lines indicate 20% of consumers, rating (−) not enough in blue and (+) too much in red.

Supportive penalty analysis provided insights into the main drivers of liking for the studied texture clusters of fruit gum candies (Figure 4), although it aggregated different products and potentially diluted product‐specific drivers. Texture attributes had high mean drops for most consumers and were therefore considered as the most important drivers of liking, with the HA cluster noted as “not being soft enough” (*p* < 0.001), while the SO cluster was described as “too soft” (*p* = 0.002). Additionally, the SO and ST clusters were perceived as “too sticky” (*p* < 0.001). The ST cluster showed mixed perceptions, as some considered it “too soft” (*p* = 0.002) whereas others found it “not soft enough” (*p* < 0.001). The CH cluster performed well in most categories, being rated as “just about right” (*p* < 0.001). Most samples were considered too sweet (several mean drops > 1, *p* < 0.001), except the HA cluster (mean drop “too sweet” 0.9, *p* = 0.035), which contained one sample with polyol as sweetener and the ST cluster (mean drop “too sweet” 0.5, *p* = 0.28), which contained the reduced sugar sample. The aroma of all presented samples was weak (all mean drops > 1, *p* < 0.05) and samples were rated as not fruity enough (all mean drops > 1.3, *p* < 0.001). For taste, samples from the HA cluster were described as “too weak” (*p* < 0.001), while the SO cluster was considered “too intense” (*p* < 0.001). Ratings for the ST cluster were divided, whereby some consumers responded that the taste was “not strong enough”, while others found it “satisfactory” (*p* < 0.001).

#### 3.3.3 Influence of Sociodemographic Factors on Overall Liking

A LMM was fitted to investigate the influence of gelling agent and consumer characteristics (age, sex) on overall liking. The results are exploratory due to the limited sample size per gelling agent.

**FIGURE 5:**
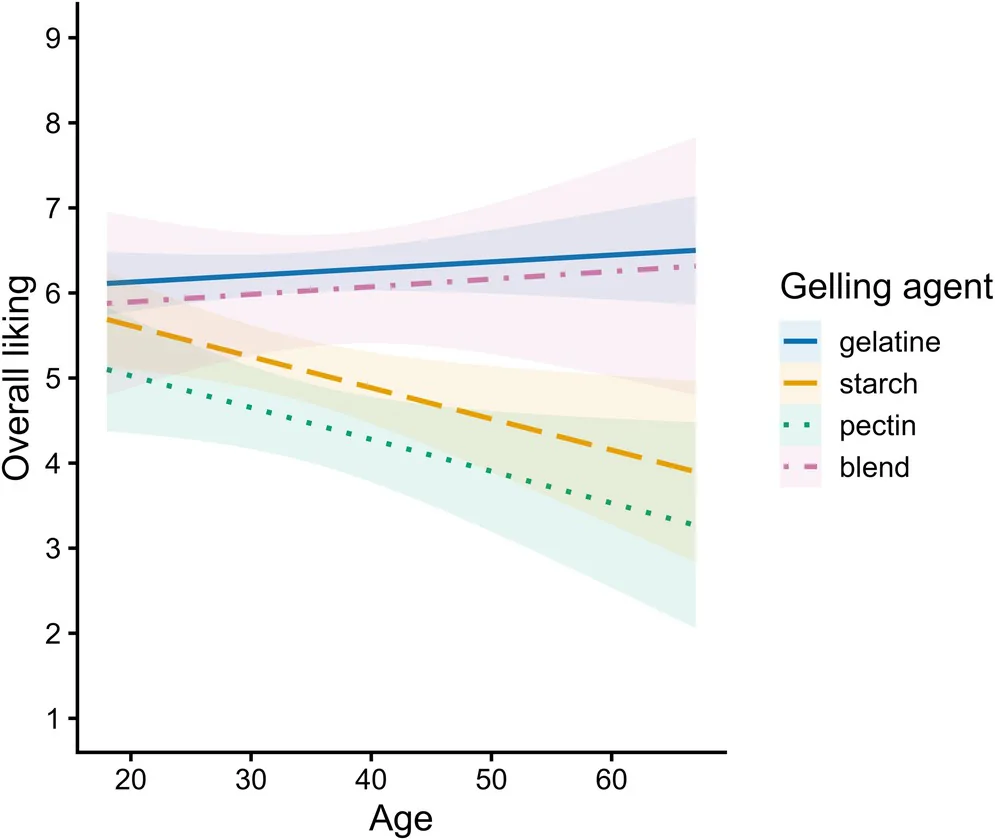
Interaction between age (18–67 years) and overall liking (nine‐point hedonic scale) with linear regression fits and 95% confidence bands, stratified by gelling agent (gelatine, starch, pectin, blend). Colors and line types denote gelling agents. Estimates are exploratory due to limited sample size.

There was a significant interaction for gelling agent with age (F_3,493_ = 4.56, *p* = 0.004) (Figure 5). Overall liking declined with increasing age for pectin (*β* = −0.04 per year, *p* = 0.006, 95% CI [−0.07, −0.01]) and starch (*β* = −0.03 per year, *p* = 0.016, 95% CI [−0.06, −0.01]), while liking of gelatine‐based samples and blend showed no significant age trend. Post hoc tests revealed no significantly higher liking for gelatine samples at mean age compared to pectin samples (t_6_ = 2.94, *p* = 0.114) or to starch (t_6_ = 3.02, *p* = 0.121). Furthermore, different evaluation behavior by sex was observed, as men rated products higher on average (*M*_Women_ = 5.46 ± 2.33, *M*_Men_ = 5.92 ± 2.18, F_1,143_ = 4.73, *p* = 0.031). There was no interaction between gelling agent and sex in rating behavior.

## 4 Discussion

Fruit gum candies are popular confectionery products among adults and children alike. Recent innovations in their formulations to develop healthier snacks are diverse and present considerable challenges regarding consumer acceptance, as modifications to the ingredients often result in altered sensory characteristics. The objectives of this study were (i) to explore sensory perception and consumer acceptance of different fruit gum candies commercially available on the German market—including both conventional and plant‐based varieties, (ii) to determine leading drivers of acceptance and the relationship between liking of single attributes and overall liking, and (iii) to examine consumer acceptance across various consumer groups.

### 4.1 Consumer Acceptance of Fruit Gum Candies

Given the dominance and high familiarity of gelatine‐based fruit gum candies on the German market, soft gelatine‐based fruit gums received higher liking ratings compared to their plant‐based counterparts (Imeson 2011; Kälviäinen et al. 2000). None of the samples received particularly high ratings; the highest rating was “like moderately” (7) for a gelatine‐based sample. Most samples were evaluated between “neither like nor dislike” (5) and “like slightly” (6). Three of the 12 samples were rated below “neither like nor dislike” (5) on the dislike end of the hedonic scale; these were all plant‐based products. Among consumers, only 39.5% reported eating fruit gum candies regularly (greater than “once a month”), which could have led to a more negative evaluation due to deviations from the typical consumption patterns of individual participants.

Not all sensory modalities are equally important for overall liking (Andersen et al. 2019). Consumers do not pay equal attention to all modalities when evaluating overall liking. This study demonstrated that within the fruit gum candy category, the liking of texture, especially hardness, plays an important role for overall liking alongside the liking of taste. Overall liking depended on all hedonic modalities except orthonasal aroma. This finding aligns with previous studies across different food categories (Andersen et al. 2019; Collier et al. 2025; Moskowitz and Krieger 1995).

### 4.2 Sensory Profile and Drivers of Liking

CATA was applied to capture sensory differences between the samples. This resulted in a correspondence analysis (CA) plot with four clusters of fruit gum candies, primarily discriminated by texture attributes. These clusters broadly correspond to the type of gelling agent used: the CH and HA clusters for gelatine‐based samples and the SO and ST clusters for plant‐based samples. By comparison, the plant‐based samples in the SO and ST clusters did not show a significant separation. These findings align with those of Obas et al. (2021), who investigated the sensory properties of various fruit gum candies. The discussion will focus on the gelling agent in terms of its dominant sensory texture perception. It is important to note that texture is also influenced by other ingredients and their proportions used in the formulation (Burey et al. 2009).

Consistent with the liking of individual sensory attributes and their influence on overall liking, the JAR ratings demonstrated that deviations in texture (stickiness, hardness) and fruitiness strongly affected overall liking, depending on the gelling agent. Increased perceived stickiness reduced liking of starch‐ and pectin‐based samples, whereas gelatine samples were least often evaluated as sticky. As perceived hardness increased, acceptance for gelatine‐based samples decreased, but increased for starch‐ or pectin‐based samples. These patterns are in line with different fracture behaviors, cohesiveness, which consequently shape oral consumption behavior. Oral consumption of fruit gum candies by chewing or sucking, for instance, and the ease with which these consumption patterns can be followed depend on their composition. Acceptance is highest when texture aligns with the preferred chewing behavior of consumers (Jeltema et al. 2015; Kamei et al. 2024).

The gel structure not only affected texture but also fruitiness and sweetness perception, as similarly observed in other studies (Gunes et al. 2022). In general, liking for all samples increased with higher JAR ratings of aroma and fruitiness. This effect was most pronounced for the attribute fruitiness and the gelling agent starch. Gelling agents and other matrix compounds exhibit different binding behaviors for flavor compounds and indirectly influence release through textural and structural properties (Pizzoni et al. 2015). Gel firmness, for example, influences the consumption process like the chewing speed or the saliva production, which consequently results in different flavor release (Boland 2004; Boland et al. 2006; Buettner and Beauchamp 2010). Softer gels are often perceived as having a stronger overall flavor due to their tendency for rapid disintegration and concomitant release of compounds. Stronger and cohesive gels retain these compounds more effectively (Déléris et al. 2011; Saint‐Eve et al. 2011).

For the softer samples from the SO cluster, this effect was only seen for the sweetness perception, not for fruitiness perception. In fruit gum candies mainly sucrose, glucose syrup, and glucose‐fructose syrup serve as sweeteners and additional stabilizers for the gel structure (Burey et al. 2009). In most selected samples, the sugar content was around 50%–55%. Among the complete sample set of 30 fruit gum candies, the majority contained sugar, with only three incorporating polyols and two having a reduced sugar content. Sweetness perception depends on the release of sweeteners into the oral cavity. Boland et al. (2006) reported that not only aroma intensities decline with increasing rigidity, but also taste intensities. This could explain the rating for the SO cluster, as the two pectin samples may release the sugar more easily than the other gel structures. Taking pectin as an example, it can require high amounts of sugar for gelation, depending on the degree of methylation of the side chains but releases sugar and flavors more rapidly because of its weaker gel structure (Boland et al. 2006; Déléris et al. 2011; Hansson et al. 2003). When combined with gelatine, pectin can create a gummy texture that is less chewy and more brittle than gelatine, while enhancing the perception of flavor and sweetness (DeMars and Ziegler 2001). Additionally, for all candies, the aroma was perceived as too weak, likely due to the evaluation of only one fruit gum at a time. Therefore, increasing JAR ratings for aroma led to higher acceptance ratings.

### 4.3 Influence of Sociodemographic Factors on Acceptance

Age had an impact on overall liking, whereby the present study revealed a significant decrease in liking for plant‐based fruit gum candies with increasing age. Kälviäinen et al. (2000) reported a similar behavior, with middle‐aged consumers showing diverse preference segmentation and younger consumer groups accepting a wider range of samples. Additionally, age can affect oro‐sensory functions, leading to changes in acceptance. Factors like bite force, oral capacity, dental health, muscle activity in the jaw and saliva production may all contribute to these changes (Liu et al. 2022). This evaluation may be further influenced by previous experience. Older participants tended to prefer gelatine‐based gums, likely because these products shaped their taste over a longer period, whereas younger participants may already be exposed to a wider variety of plant‐based fruit gum candies as these products have evolved in recent years, shaping their preferences from a young age. Furthermore, younger individuals might be more inclined to experiment with new products, potentially due to increased environmental awareness and acceptance of plant‐based diets (Kälviäinen et al. 2000; Lehmann et al. 2025). Regular consumption of these alternatives may also enhance acceptance of diverse food options through repeated exposure (Schicker et al. 2023). Given the small observed effect for age, these interpretations should be viewed with caution.

Men rated samples slightly higher than women. This observation aligns with the results of other studies that showed differences in the evaluation behavior with regard to sex (Kälviäinen et al. 2000). Specifically, it was observed that females preferred short and fracturing candy textures, while males preferred harder and more adhesive textures. Another study revealed that sensitivity to hardness slightly influenced the preference for different fruit gum candies, highlighting that participant characteristics, like sex and age, can significantly impact the perception of hardness and food acceptance (Puleo et al. 2021). In general, however, understanding texture sensitivity and its relationship to texture acceptance remains challenging due to its multidimensional and dynamic nature (Liu et al. 2022).

### 4.4 Study Limitations

The primary objective of this study was to capture consumer acceptance for various fruit gum candies in relation to specific sensory properties. The use of commercial products imposes limits on drawing conclusions on causality between the ingredients and the sensory properties. Information on several influencing factors was not available on the product labelling, like the origin and type of gelling agent or the ratios of the ingredients in the formulation. Potential effects of differences in origin or type of gelling agent (e.g., porcine or bovine gelatine, different esterification of pectin) on texture properties and consumer acceptance could therefore not be systematically evaluated. The same applies to the influence of the fruit juice content, which varied between 0% and 30%. Furthermore, the selection of samples for the consumer study was made based on sensory properties and is only partially representative of the individual ingredient classes (e.g., different sweeteners). Future research should therefore include controlled formulation studies in which the type, origin and concentration of gelling agents, sweeteners and other ingredients are systematically varied in standardized fruit gum matrices and combined with instrumental texture and physicochemical measurements to allow a more precise evaluation of the effects of ingredients on sensory perception and consumer acceptance. Additionally, the commercial samples contained a mixture of different flavors and colors in one package, which introduces within‐pack variability in these sensory impressions for color, aroma, fruitiness and liking. Related questions posed to the naïve panel were therefore kept as general as possible, as consumers tend to consume a mixture of fruit gum candies from a package and not just one color or flavor.

The incomplete design affects the discriminatory power of the consumer study. The study aimed to evaluate as many different samples as possible without overwhelming consumers, which resulted in an unbalanced, incomplete design randomized across the texture clusters. The assumption that samples are similar in their sensory properties and could therefore be grouped for randomization for the incomplete presentation to consumers underestimated the nuances of sensory properties and acceptance ratings.

Specific criteria limited the sample population as the study was conducted at a university event predominantly attended by relatively young visitors. Future studies should include a larger and more heterogeneous population in terms of age, as well as income and education (no related information collected in this study). Additionally, the study recruited predominantly German consumers, but since fruit gum candy confectioneries are enjoyed worldwide, conducting similar studies in different countries would provide insights into the preferences and acceptance of different consumer groups.

The parameters of the study enforced the use of a simpler design that focussed solely on acceptance, nine‐point hedonic scales and drivers of liking through traditional JAR questions. There is potential for expansion in terms of exploring associations, free comments or emotions that influence acceptance. As texture is one of the most discriminating sensory modalities, further refinement of the sensory attributes related to texture is needed for better characterization. A more descriptive approach may be preferrable to the CATA evaluation employed in this study. Additionally, targeting specific consumer groups (e.g., regular users) or consumption patterns (e.g., adhering to a plant‐based diet) could provide insights into how dietary habits influence acceptance. In this study, 13.6% of participants identified as vegetarian or vegan, indicating insufficient numbers for providing general effects for this specific consumer group. Nevertheless, their acceptance may be influenced by repeated exposure to different plant‐based products (Schicker et al. 2023). Moreover, further exploration of chewing behavior (e.g., whether participants chew or suck on the fruit gum candies) is warranted, as these behaviors are closely related to acceptance.

## 5 Conclusion

The results of the present study reveal that acceptance of fruit gum candies is primarily related to taste and texture liking. In particular, stickiness and hardness were decisive for consumer acceptance of plant‐based fruit gum candies; thus, such candies can narrow the acceptance gap to gelatine‐based versions by reducing stickiness and increasing hardness while enhancing fruitiness and aroma. Furthermore, younger consumers rated plant‐based fruit gum candies higher, while older consumers preferred gelatine‐based versions.

This study highlights the relationship between choice of gelling agent, product characteristics, sensory perception and consumer acceptance of fruit gum candies, especially those made with plant‐based gelling agents. As plant‐based formulations become increasingly prevalent in the market, understanding these relationships helps to develop products that meet consumer expectations. The sensory characteristics and consumer acceptance data from the present study could be used to further tailor alternative products to optimal sensory properties. As texture perception is a complex process, a more detailed evaluation of different consumption steps may be necessary, and an expanded texture evaluation could improve discrimination of samples.

## Author Contributions

**Maria Izaber‐Ludwig:** conceptualization, data curation, investigation, formal analysis, visualization, writing – original draft, writing – review and editing. **Zinaida Catruc:** writing – review and editing, investigation, visualization, formal analysis, conceptualization. **Jonathan Beauchamp:** writing – review and editing, conceptualization, supervision. **Jessica Freiherr:** conceptualization, supervision, writing – review and editing.

## Funding

This work was supported by the Federal Ministry for Economic Affairs and Energy (Bundesministerium für Wirtschaft und Energie [BMWE]) under the frame of industrial collaborative research program within the project “CandyCrunch” (funding reference number: 01IF00332C).

## Supporting information


**Figure S1:** Contingency table of the selected attributes across all 30 fruit gum candies, depicted as a heatmap. Colors transition from blue (0; no mentions) over white (5) to red (10; chosen by all panelists); samples are ordered according to categorization from the agglomerative hierarchical cluster (AHC) analysis: SO—soft texture, ST—sticky texture, CH—chewy texture, HA—hard texture.
**Figure S2:** Dendrogram from the agglomerative hierarchical cluster (AHC) on check‐all‐that‐apply (CATA) data, revealing four clusters (C1‐4) across 30 products (P1‐P30).
**Table S1:** Values for Hartigan index (H) and the difference between different number of clusters (k). The bold value indicates the largest difference and suggests four clusters.
**Table S2:** Mean consumer hedonic ratings (M) and standard deviations (SD) for fruit gum candy samples (*n* = 12). Participants were asked to rate how much they like the indicated attribute on a fully labeled 9‐point category scale from “dislike extremely” (1) to “like extremely” (9). Letters indicate significant difference in pairwise comparisons within a sensory modality (*p* < 0.05).
**Table S3:** Results of linear mixed models to evaluate overall liking, liking of color, aroma and taste.
**Table S4:** Results of linear mixed models to evaluate liking of sweetness, fruitiness, stickiness and hardness.
**Table S5:** Results of the linear mixed model to evaluate influence of liking of individual sensory attributes on overall liking.
**Table S6:** Mean (M) just‐about‐right (JAR) ratings with standard deviation (SD) clustered with comparable properties. Participants were asked to rate attributes on a fully labeled 5‐point category scale from “not enough” (1) to “too much” (5).
**Table S7:** Results of the linear mixed model to evaluate influence of centred just‐about‐right (JAR) ratings of sensory attributes on overall liking.
**Table S8:** Trends of the different sensory attributes (just‐about‐right; JAR) in relation to consumer acceptance based on linear mixed model.
**Table S9:** Results of the linear mixed model to evaluate influence of ingredients and sociodemographic factors on overall liking.

## Data Availability

The data that support the findings of this study are available from the corresponding author upon reasonable request.
