# Supplementary material for: Texture Matters: Linking Sensory Properties of Fruit Gum Candies to Consumer Acceptance
Source: Food Sci Nutr. 2026 Aug 24;14(8):e72267. doi: 10.1002/fsn3.72267 (PMC13504158; doi:10.1002/fsn3.72267)
Supplement: Supplementary file 1 [file FSN3-14-e72267-s001.docx]

**Texture Matters: Linking Sensory Properties of Fruit Gum Candies to Consumer Acceptance**

Maria Izaber-Ludwig^1,2^, Zinaida Catruc^1^, Jonathan Beauchamp^1^, Jessica Freiherr^1,2^

^1^Department of Sensory Analytics and Technologies, Fraunhofer Institute for Process Engineering and Packaging IVV, 85354 Freising, Germany

^2^Department of Psychiatry and Psychotherapy, Friedrich-Alexander-Universität Erlangen-Nürnberg, 91054 Erlangen, Germany

Corresponding authors: [maria.izaber-ludwig@ivv.fraunhofer.de /](rewritten://3db8fbc6-19f1-43a5-ba32-9c047a8d46f5) [jessica.freiherr@ivv.fraunhofer.de](mailto:jessica.freiherr@ivv.fraunhofer.de)

**- Supplementary Material –**

## 1 Remarks

Linear mixed model (LMM) descriptions were created using the *report* package in R (Makowski et al., 2021).

## 2  Sensory Evaluation – Check-all-that-Apply

**Figure S1.** Contingency table of the selected attributes across all 30 fruit gum candies, depicted as a heatmap. Colors transition from blue (0; no mentions) over white (5) to red (10; chosen by all panelists); samples are ordered according to categorization from the agglomerative hierarchical cluster (AHC) analysis: SO – soft texture, ST – sticky texture, CH – chewy texture, HA – hard texture.

**Figure S2.** Dendrogram from the agglomerative hierarchical cluster (AHC) on check-all-that-apply (CATA) data, revealing four clusters (C1-4) across 30 products (P1-P30).

**Table S1.** Values for Hartigan index (H) and the difference between different number of clusters (k). The bold value indicates the largest difference and suggests four clusters.

| Indices | Number of clusters | | | |
| --- | --- | --- | --- | --- |
|  | 2 | 3 | 4 | 5 |
| Hartigan index (H) | 13.395 | 13.337 | 3.313 | 3.526 |
| H(k-1) - H(k) | 3.188 | 0.058 | **10.024** | -0.213 |

## 3 Sensory Evaluation – Consumer Study

**Table S2.** Mean consumer hedonic ratings (M) and standard deviations (SD) for fruit gum candy samples (*n* = 12). Participants were asked to rate how much they like the indicated attribute on a fully labelled 9-point category scale from “dislike extremely” (1) to “like extremely” (9). Letters indicate significant difference in pairwise comparisons within a sensory modality (*p* < 0.05).

| Texture cluster^1^ | Sample | *n* | Over-all liking (OL) |  | Gr. OL | Color |  | Gr. Color | Aroma |  | Gr. Aroma | Taste |  | Gr. Taste | Sweet-ness |  | Gr. Sweet-ness | Fruiti-ness |  | Gr.  Fruiti-ness | Sticki-ness |  | Gr. Sticki-ness | Hard-ness |  | Gr. Hard-ness |
| --- | --- | --- | --- | --- | --- | --- | --- | --- | --- | --- | --- | --- | --- | --- | --- | --- | --- | --- | --- | --- | --- | --- | --- | --- | --- | --- |
|  |  |  | *M* | *SD* |  | *M* | *SD* |  | *M* | *SD* |  | *M* | *SD* |  | *M* | *SD* |  | *M* | *SD* |  | *M* | *SD* |  | *M* | *SD* |  |
| SO | P18 | 51 | 5.45 | 2.33 | bcd | 6.45 | 1.99 | abc | 5.51 | 2.04 | ab | 5.84 | 2.61 | bcd | 6.16 | 2.24 | abcd | 6.20 | 2.18 | ab | 4.33 | 2.52 | ab | 4.77 | 2.45 | abc |
|  | P21 | 42 | 4.17 | 2.17 | a | 6.19 | 2.46 | abc | 5.00 | 1.85 | a | 4.62 | 2.35 | a | 5.02 | 2.16 | a | 4.91 | 2.55 | a | 3.21 | 2.09 | a | 5.05 | 2.48 | abc |
|  | P30 | 54 | 4.74 | 2.43 | ab | 5.94 | 2.11 | abc | 6.22 | 2.16 | ab | 5.69 | 2.64 | abc | 5.56 | 2.17 | ab | 6.11 | 2.21 | ab | 4.57 | 2.55 | ab | 4.00 | 2.54 | a |
| ST | P12 | 47 | 4.70 | 2.17 | ab | 5.57 | 2.19 | a | 5.83 | 2.10 | ab | 5.15 | 2.43 | abc | 5.62 | 2.60 | abc | 5.28 | 2.12 | a | 3.34 | 2.40 | a | 4.30 | 2.40 | abc |
|  | P13 | 56 | 5.25 | 2.40 | abcd | 5.71 | 2.35 | a | 5.45 | 1.86 | a | 5.36 | 2.51 | abc | 5.86 | 2.02 | abc | 5.46 | 2.31 | a | 4.84 | 2.54 | bc | 5.61 | 2.27 | bcd |
|  | P24 | 44 | 6.05 | 2.11 | cdef | 6.09 | 1.85 | abc | 5.55 | 1.86 | ab | 6.36 | 2.01 | cde | 6.05 | 2.03 | abcd | 6.23 | 2.19 | ab | 5.36 | 2.39 | bcd | 5.66 | 2.37 | bcd |
| CH | P4 | 34 | 6.62 | 1.81 | def | 6.94 | 2.00 | bc | 7.00 | 1.33 | b | 6.77 | 1.96 | cde | 7.21 | 1.63 | cd | 7.06 | 2.16 | c | 6.56 | 2.11 | de | 6.06 | 2.40 | cd |
|  | P19 | 62 | 6.95 | 1.57 | ef | 6.89 | 1.57 | bc | 6.55 | 1.82 | b | 6.92 | 1.64 | de | 6.71 | 1.76 | cd | 6.92 | 1.83 | c | 6.79 | 1.83 | e | 6.89 | 1.87 | d |
|  | P28 | 51 | 7.29 | 1.39 | f | 7.10 | 1.19 | c | 6.26 | 1.93 | ab | 7.43 | 1.46 | e | 7.14 | 1.58 | d | 7.28 | 1.58 | c | 6.86 | 1.66 | e | 6.77 | 1.95 | d |
| HA | P1 | 52 | 5.33 | 2.21 | abcd | 6.48 | 1.74 | abc | 6.25 | 1.86 | ab | 6.00 | 2.27 | bcde | 6.44 | 1.81 | bcd | 5.94 | 2.05 | ab | 6.08 | 1.80 | cde | 4.27 | 2.76 | ab |
|  | P5 | 45 | 5.29 | 2.14 | abcd | 5.73 | 1.97 | ab | 5.53 | 2.11 | ab | 5.07 | 2.31 | ab | 5.96 | 2.18 | abcd | 5.38 | 2.26 | a | 5.76 | 2.18 | bcde | 4.56 | 2.56 | abc |
|  | P7 | 50 | 5.82 | 2.23 | bcde | 6.22 | 2.14 | abc | 5.84 | 1.99 | ab | 6.48 | 1.73 | bcde | 6.48 | 1.89 | bcd | 6.02 | 1.81 | ab | 6.10 | 2.09 | cde | 4.58 | 2.57 | abc |

^1^SO = soft texture, ST = sticky texture, CH = chewy texture, HA = hard texture; Note: Multiple comparisons were done using *emmeans* and *multcomp* packages in R (Hothorn et al., 2002; Lenth, 2025). Identical letters within each column (Gr. = Grouping) indicate mean values that do not differ significantly in pairwise comparisons (*emmeans*; Holm correction; *p* < .05). The grouping was calculated across all samples (*n* = 12); clusters are purely descriptive from the CATA analysis.

**Table S3.** Results of linear mixed models to evaluate overall liking, liking of color, aroma and taste.

|  | **Overall liking** | | | | **Color** | | | | **Aroma** | | | | **Taste** | | | |
| --- | --- | --- | --- | --- | --- | --- | --- | --- | --- | --- | --- | --- | --- | --- | --- | --- |
| *Predictors* | *Estimates* | *std. Error* | *CI* | *p* | *Estimates* | *std. Error* | *CI* | *p* | *Estimates* | *std. Error* | *CI* | *p* | *Estimates* | *std. Error* | *CI* | *p* |
| Intercept | 5.43 | 0.29 | 4.86 – 6.00 | **<0.001** | 6.54 | 0.27 | 6.00 – 7.08 | **<0.001** | 6.30 | 0.27 | 5.78 – 6.82 | **<0.001** | 6.16 | 0.30 | 5.58 – 6.74 | **<0.001** |
| P5 | -0.19 | 0.42 | -1.02 – 0.64 | 0.651 | -0.85 | 0.40 | -1.63 – -0.06 | **0.035** | -0.81 | 0.39 | -1.57 – -0.04 | **0.038** | -1.19 | 0.43 | -2.02 – -0.35 | **0.006** |
| P7 | 0.33 | 0.41 | -0.48 – 1.13 | 0.424 | -0.34 | 0.39 | -1.11 – 0.42 | 0.378 | -0.47 | 0.38 | -1.22 – 0.27 | 0.211 | 0.23 | 0.41 | -0.58 – 1.05 | 0.578 |
| P12 | -0.72 | 0.41 | -1.53 – 0.09 | 0.080 | -0.96 | 0.39 | -1.73 – -0.19 | **0.014** | -0.46 | 0.38 | -1.21 – 0.29 | 0.226 | -1.00 | 0.42 | -1.82 – -0.19 | **0.016** |
| P13 | -0.23 | 0.39 | -1.00 – 0.54 | 0.556 | -0.88 | 0.37 | -1.61 – -0.14 | **0.019** | -0.90 | 0.36 | -1.62 – -0.19 | **0.013** | -0.87 | 0.39 | -1.64 – -0.10 | **0.028** |
| P24 | 0.67 | 0.41 | -0.14 – 1.48 | 0.105 | -0.38 | 0.40 | -1.16 – 0.39 | 0.334 | -0.70 | 0.39 | -1.46 – 0.06 | 0.070 | 0.27 | 0.42 | -0.54 – 1.09 | 0.512 |
| P18 | 0.12 | 0.40 | -0.67 – 0.91 | 0.765 | -0.05 | 0.38 | -0.80 – 0.70 | 0.903 | -0.76 | 0.37 | -1.49 – -0.03 | **0.042** | -0.16 | 0.40 | -0.96 – 0.63 | 0.686 |
| P21 | -1.37 | 0.42 | -2.20 – -0.55 | **0.001** | -0.37 | 0.40 | -1.16 – 0.42 | 0.363 | -1.31 | 0.39 | -2.08 – -0.54 | **0.001** | -1.62 | 0.43 | -2.46 – -0.79 | **<0.001** |
| P30 | -0.70 | 0.39 | -1.47 – 0.07 | 0.077 | -0.62 | 0.38 | -1.36 – 0.12 | 0.100 | -0.10 | 0.37 | -0.82 – 0.62 | 0.782 | -0.56 | 0.40 | -1.34 – 0.22 | 0.156 |
| P4 | 1.12 | 0.45 | 0.24 – 2.01 | **0.013** | 0.41 | 0.43 | -0.43 – 1.25 | 0.339 | 0.61 | 0.42 | -0.21 – 1.43 | 0.144 | 0.52 | 0.45 | -0.38 – 1.41 | 0.256 |
| P19 | 1.53 | 0.38 | 0.78 – 2.28 | **<0.001** | 0.34 | 0.36 | -0.37 – 1.06 | 0.349 | 0.29 | 0.35 | -0.40 – 0.99 | 0.406 | 0.79 | 0.38 | 0.04 – 1.55 | **0.039** |
| P28 | 1.90 | 0.40 | 1.11 – 2.68 | **<0.001** | 0.56 | 0.38 | -0.19 – 1.31 | 0.141 | -0.04 | 0.37 | -0.77 – 0.69 | 0.907 | 1.28 | 0.40 | 0.49 – 2.07 | **0.001** |
| **Random Effects** |  |  |  |  |  |  |  |  |  |  |  |  |  |  |  |  |
| σ^2^ | 3.83 | | | | 3.56 | | | | 3.37 | | | | 3.80 | | | |
| τ_00_ | 0.59 _Participant_ | | | | 0.37 _Participant_ | | | | 0.36 _Participant_ | | | | 1.01 _Participant_ | | | |
| ICC | 0.13 | | | | 0.09 | | | | 0.10 | | | | 0.21 | | | |
| N | 147 _Participant_ | | | | 147 _Participant_ | | | | 147 _Participant_ | | | | 147 _Participant_ | | | |
| Observations | 588 | | | | 588 | | | | 588 | | | | 588 | | | |
| Marginal R^2^ / Conditional R^2^ | 0.162 / 0.275 | | | | 0.060 / 0.148 | | | | 0.065 / 0.155 | | | | 0.125 / 0.308 | | | |

Note: Data was analyzed with linear mixed models (estimated using REML and nloptwrap optimizer) for each sensory modality (formula: respective liking ratings ~ sample). The model included participant as random effect (formula: ~1 |participant). The model’s intercept corresponds to sample P1. Standardized parameters were obtained by fitting the models on a standardized version of the dataset. 95 % confidence intervals (CIs) and *p*-values were computed using Kenward–Roger degrees of freedom. ICC = Intraclass Correlation Coefficient; τ_00_ denotes the variance in intercepts, σ^2^ denotes the residual variance

**Table S4.** Results of linear mixed models to evaluate liking of sweetness, fruitiness, stickiness and hardness.

|  | **Sweetness** | | | | **Fruitiness** | | | | **Stickiness** | | | | **Hardness** | | | |
| --- | --- | --- | --- | --- | --- | --- | --- | --- | --- | --- | --- | --- | --- | --- | --- | --- |
| *Predictors* | *Estimates* | *std. Error* | *CI* | *p* | *Estimates* | *std. Error* | *CI* | *p* | *Estimates* | *std. Error* | *CI* | *p* | *Estimates* | *std. Error* | *CI* | *p* |
| Intercept | 6.47 | 0.28 | 5.92 – 7.01 | **<0.001** | 6.04 | 0.29 | 5.48 – 6.61 | **<0.001** | 6.09 | 0.31 | 5.49 – 6.69 | **<0.001** | 4.27 | 0.33 | 3.62 – 4.92 | **<0.001** |
| P5 | -0.55 | 0.40 | -1.33 – 0.24 | 0.171 | -0.72 | 0.42 | -1.55 – 0.11 | 0.088 | -0.33 | 0.45 | -1.21 – 0.55 | 0.462 | 0.29 | 0.49 | -0.67 – 1.25 | 0.552 |
| P7 | 0.03 | 0.39 | -0.74 – 0.79 | 0.947 | -0.08 | 0.41 | -0.88 – 0.73 | 0.846 | -0.00 | 0.44 | -0.86 – 0.86 | 0.997 | 0.31 | 0.47 | -0.62 – 1.24 | 0.517 |
| P12 | -0.84 | 0.39 | -1.60 – -0.07 | **0.032** | -0.72 | 0.41 | -1.52 – 0.09 | 0.082 | -2.76 | 0.44 | -3.63 – -1.89 | **<0.001** | 0.03 | 0.48 | -0.92 – 0.97 | 0.955 |
| P13 | -0.70 | 0.37 | -1.43 – 0.03 | 0.059 | -0.68 | 0.39 | -1.44 – 0.09 | 0.085 | -1.26 | 0.42 | -2.09 – -0.43 | **0.003** | 1.33 | 0.46 | 0.43 – 2.23 | **0.004** |
| P24 | -0.31 | 0.39 | -1.08 – 0.46 | 0.426 | 0.25 | 0.41 | -0.56 – 1.07 | 0.540 | -0.70 | 0.45 | -1.58 – 0.18 | 0.121 | 1.40 | 0.49 | 0.44 – 2.36 | **0.004** |
| P18 | -0.27 | 0.38 | -1.01 – 0.48 | 0.482 | 0.21 | 0.40 | -0.58 – 1.00 | 0.601 | -1.74 | 0.43 | -2.59 – -0.89 | **<0.001** | 0.51 | 0.47 | -0.42 – 1.43 | 0.283 |
| P21 | -1.47 | 0.40 | -2.26 – -0.69 | **<0.001** | -1.18 | 0.42 | -2.01 – -0.35 | **0.005** | -2.89 | 0.45 | -3.78 – -2.00 | **<0.001** | 0.77 | 0.50 | -0.20 – 1.74 | 0.120 |
| P30 | -0.92 | 0.37 | -1.65 – -0.19 | **0.013** | 0.05 | 0.39 | -0.73 – 0.82 | 0.902 | -1.52 | 0.43 | -2.35 – -0.68 | **<0.001** | -0.27 | 0.46 | -1.18 – 0.64 | 0.556 |
| P4 | 0.62 | 0.43 | -0.22 – 1.45 | 0.150 | 0.93 | 0.45 | 0.05 – 1.82 | **0.039** | 0.48 | 0.48 | -0.47 – 1.43 | 0.322 | 1.79 | 0.53 | 0.76 – 2.83 | **0.001** |
| P19 | 0.29 | 0.36 | -0.42 – 1.00 | 0.416 | 0.91 | 0.38 | 0.16 – 1.66 | **0.017** | 0.70 | 0.41 | -0.11 – 1.51 | 0.090 | 2.61 | 0.45 | 1.73 – 3.50 | **<0.001** |
| P28 | 0.70 | 0.38 | -0.05 – 1.44 | 0.066 | 1.25 | 0.40 | 0.46 – 2.03 | **0.002** | 0.77 | 0.43 | -0.08 – 1.62 | 0.074 | 2.50 | 0.47 | 1.58 – 3.42 | **<0.001** |
| **Random Effects** | | | | | | | | | | | | | | | | |
| σ^2^ | 3.40 | | | | 3.85 | | | | 4.70 | | | | 5.62 | | | |
| τ_00_ | 0.69 _Participant_ | | | | 0.58 _Participant_ | | | | 0.13 _Participant_ | | | | 0.07 _Participant_ | | | |
| ICC | 0.17 | | | | 0.13 | | | | 0.03 | | | | 0.01 | | | |
| N | 147 _Participant_ | | | | 147 _Participant_ | | | | 147 _Participant_ | | | | 147 _Participant_ | | | |
| Observations | 588 | | | | 588 | | | | 588 | | | | 588 | | | |
| Marginal R^2^ / Conditional R^2^ | 0.082 / 0.236 | | | | 0.102 / 0.220 | | | | 0.229 / 0.250 | | | | 0.142 / 0.153 | | | |

Note: Data was analyzed with linear mixed models (estimated using REML and *nloptwrap* optimizer) for each sensory modality (formula: respective liking ratings ~ sample). The model included participant as random effect (formula: ~1 |participant). The model’s intercept corresponds to sample P1. Standardized parameters were obtained by fitting the models on a standardized version of the dataset. 95 % confidence intervals (CIs) and *p*-values were computed using Kenward–Roger degrees of freedom. ICC = Intraclass Correlation Coefficient; τ_00_ denotes the variance in intercepts, σ^2^ denotes the residual variance.

**Table S5.** Results of the linear mixed model to evaluate influence of liking of individual sensory attributes on overall liking.

|  | **Overall liking** | | | |
| --- | --- | --- | --- | --- |
| *Predictors* | *Estimates* | *std. Error* | *CI* | *p* |
| Intercept | -0.71 | 0.20 | -1.11 – -0.32 | **<0.001** |
| Liking of Color | 0.06 | 0.03 | 0.01 – 0.12 | **0.022** |
| Liking of Aroma | 0.02 | 0.03 | -0.04 – 0.07 | 0.547 |
| Liking of Taste | 0.36 | 0.03 | 0.30 – 0.43 | **<0.001** |
| Liking of Sweetness | 0.10 | 0.03 | 0.03 – 0.16 | **0.003** |
| Liking of Fruitiness | 0.17 | 0.03 | 0.11 – 0.23 | **<0.001** |
| Liking of Hardness | 0.24 | 0.02 | 0.20 – 0.29 | **<0.001** |
| Liking of Stickiness | 0.15 | 0.03 | 0.10 – 0.20 | **<0.001** |
| **Random Effects** | | | | |
| σ^2^ | 1.10 | | | |
| τ_00_ _Participant_ | 0.15 | | | |
| τ_00_ _Sample_ | 0.01 | | | |
| ICC | 0.13 | | | |
| N _Participant_ | 147 | | | |
| N _Sample_ | 12 | | | |
| Observations | 588 | | | |
| Marginal R^2^ / Conditional R^2^ | 0.758 / 0.789 | | | |

Note: Data was analyzed with a linear mixed model (estimated using REML and *nloptwrap* optimizer) to predict Overall liking with liking of color, aroma, taste, sweetness, fruitiness, hardness and stickiness (formula: Overall liking ~ liking of color + liking of aroma+ liking of taste + liking of sweetness + liking of fruitiness + liking of hardness + liking of stickiness). The model included participant and samples as random effects (formula: ~1 | participant, ~1 | sample). The model’s intercept corresponds to liking of color. Standardized parameters were obtained by fitting the models on a standardized version of the dataset. 95 % confidence intervals (CIs) and *p*-values were computed using Kenward–Roger degrees of freedom. ICC = Intraclass Correlation Coefficient; τ_00_ denotes the variance in intercepts, σ^2^ denotes the residual variance.

**Table S6.** Mean (M) just-about-right (JAR) ratings with standard deviation (SD) clustered with comparable properties. Participants were asked to rate attributes on a fully labelled 5-point category scale from ‘not enough’ (1) to ‘too much’ (5).

| Texture cluster^1^ | Color |  | Aroma |  | Taste |  | Sweetness |  | Fruity |  | Stickiness |  | Softness |  |
| --- | --- | --- | --- | --- | --- | --- | --- | --- | --- | --- | --- | --- | --- | --- |
|  | *M* | *SD* | *M* | *SD* | *M* | *SD* | *M* | *SD* | *M* | *SD* | *M* | *SD* | *M* | *SD* |
| SO | 3.12 | 0.71 | 2.53 | 0.87 | 3.14 | 0.83 | 3.47 | 0.83 | 2.67 | 0.81 | 3.81 | 1.03 | 3.74 | 1.05 |
| ST | 2.61 | 0.80 | 2.58 | 0.82 | 2.84 | 0.86 | 3.10 | 0.81 | 2.52 | 0.79 | 3.92 | 0.82 | 3.11 | 0.96 |
| CH | 2.79 | 0.55 | 2.82 | 0.68 | 3.06 | 0.59 | 3.28 | 0.63 | 2.85 | 0.61 | 3.13 | 0.55 | 3.02 | 0.74 |
| HA | 2.94 | 0.81 | 2.80 | 0.77 | 2.90 | 0.80 | 3.10 | 0.73 | 2.59 | 0.79 | 2.92 | 0.70 | 2.10 | 0.89 |

^1^SO = soft texture, ST = sticky texture, CH = chewy texture, HA = hard texture

**Table S7.** Results of the linear mixed model to evaluate influence of centered just-about-right (JAR) ratings of sensory attributes on overall liking.

|  | **Overall liking** | | | |
| --- | --- | --- | --- | --- |
| *Predictors* | *Estimates* | *std. Error* | *CI* | *p* |
| Intercept | 6.82 | 0.19 | 6.40 – 7.25 | **<0.001** |
| cJAR Aroma | 0.30 | 0.11 | 0.09 – 0.52 | **0.005** |
| cJAR Taste | -0.35 | 0.12 | -0.58 – -0.12 | **0.002** |
| Gelling agent [Pectin] | -1.27 | 0.40 | -2.13 – -0.40 | **0.007** |
| Gelling agent [Starch] | 0.07 | 0.36 | -0.70 – 0.84 | 0.852 |
| Gelling agent [Blend] | 0.20 | 0.53 | -0.94 – 1.34 | 0.714 |
| cJAR Fruity | 0.50 | 0.16 | 0.18 – 0.82 | **0.002** |
| cJAR Sticky | 0.09 | 0.17 | -0.26 – 0.43 | 0.623 |
| cJAR Hard | -0.91 | 0.13 | -1.17 – -0.65 | **<0.001** |
| Gelling agent [Pectin] x cJAR Fruity | 0.12 | 0.28 | -0.43 – 0.68 | 0.664 |
| Gelling agent [Starch] x cJAR Fruity | 0.80 | 0.25 | 0.32 – 1.28 | **0.001** |
| Gelling agent [Blend] x cJAR Fruity | 0.08 | 0.42 | -0.74 – 0.90 | 0.843 |
| Gelling agent [Pectin] x cJAR Sticky | -0.46 | 0.25 | -0.94 – 0.03 | 0.065 |
| Gelling agent [Starch] x cJAR Sticky | -0.97 | 0.25 | -1.47 – -0.47 | **<0.001** |
| Gelling agent [Blend] x cJAR Sticky | -1.14 | 0.40 | -1.93 – -0.35 | **0.005** |
| Gelling agent [Pectin] x cJAR Hard | 1.50 | 0.23 | 1.04 – 1.95 | **<0.001** |
| Gelling agent [Starch] x cJAR Hard | 1.25 | 0.20 | 0.86 – 1.65 | **<0.001** |
| Gelling agent [Blend] x cJAR Hard | 0.34 | 0.32 | -0.29 – 0.98 | 0.291 |
| **Random Effects** | | | | |
| σ^2^ | 2.97 | | | |
| τ_00_ _Participant_ | 0.54 | | | |
| τ_00_ _Sample_ | 0.10 | | | |
| ICC | 0.18 | | | |
| N _Sample_ | 12 | | | |
| N _Participant_ | 147 | | | |
| Observations | 588 | | | |
| Marginal R^2^ / Conditional R^2^ | 0.318 / 0.439 | | | |

Note: Data was analyzed with a linear mixed model (estimated using REML and *nloptwrap* optimizer) to predict Overall liking with centered just about right-ratings (cJAR) for sensory attributes, gelling agent, and their specific two way interactions (final formula: Overall liking ~ cJAR aroma + cJAR taste + gelling agent *(cJAR fruity + cJAR sticky + cJAR hard)). The model included participant and samples as random effects (formula: ~1 | participant, ~1 | sample). The model’s intercept corresponds to cJARAroma = 0, cJARTaste = 0, gel_agent = gelatine, cJARFruity = 0, cJARSticky = 0 and cJARHardn = 0. Standardized parameters were obtained by fitting the models on a standardized version of the dataset. 95 % confidence intervals (CIs) and *p*-values were computed using Kenward–Roger degrees of freedom. ICC = Intraclass Correlation Coefficient; τ_00_ denotes the variance in intercepts, σ^2^ denotes the residual variance. Results for pectin and blend should be interpreted cautiously due to limited sample size. Full model was initially considered and then stepwise reduced by excluding non-significant terms.

**Table S8.** Trends of the different sensory attributes (just-about-right; JAR) in relation to consumer acceptance based on linear mixed model.

| **Sensory attribute** | **Gelling agent** | **Trend (β)** | **std. Error** | **df** | **95% CI** | ***t*-value** | ***p-*value** |
| --- | --- | --- | --- | --- | --- | --- | --- |
| Fruity | gelatine | 0.50 | 0.16 | 559 | 0.18 | 3.07 | 0.002 |
|  | pectin | 0.62 | 0.24 | 504 | 0.16, 1.09 | 2.62 | 0.009 |
|  | starch | 1.30 | 0.21 | 555 | 0.89, 1.71 | 6.19 | <0.001 |
|  | blend | 0.58 | 0.39 | 543 | -0.19, 1.35 | 1.48 | 0.139 |
| Sticky | gelatine | 0.09 | 0.17 | 569 | -0.26, 0.43 | 0.49 | 0.623 |
|  | pectin | -0.37 | 0.18 | 456 | -0.73, -0.01 | -2.05 | 0.041 |
|  | starch | -0.89 | 0.18 | 559 | -1.25, -0.52 | -4.79 | <0.001 |
|  | blend | -1.05 | 0.36 | 541 | -1.77, -0.34 | -2.89 | 0.004 |
| Hard | gelatine | -0.91 | 0.13 | 252 | -1.17, -0.65 | -6.82 | <0.001 |
|  | pectin | 0.59 | 0.19 | 373 | 0.21, 0.96 | 3.05 | 0.002 |
|  | starch | 0.34 | 0.15 | 528 | 0.04, 0.64 | 2.22 | 0.027 |
|  | blend | -0.57 | 0.30 | 544 | -1.16, 0.02 | -1.89 | 0.060 |

**Table S9.** Results of the linear mixed model to evaluate influence of ingredients and sociodemographic factors on overall liking.

|  | **Overall liking** | | | |
| --- | --- | --- | --- | --- |
| *Predictors* | *Estimates* | *std. Error* | *CI* | *p* |
| Intercept | 6.03 | 0.32 | 5.31 – 6.74 | **<0.001** |
| Gelling agent [Pectin] | -1.78 | 0.61 | -3.19 – -0.38 | **0.019** |
| Gelling agent [Starch] | -1.11 | 0.52 | -2.32 – 0.11 | 0.069 |
| Gelling agent [Blend] | -0.15 | 0.81 | -2.02 – 1.71 | 0.855 |
| Age | 0.01 | 0.01 | -0.01 – 0.03 | 0.409 |
| Sex [Male] | 0.45 | 0.21 | 0.04 – 0.85 | **0.031** |
| Gelling agent [Pectin] x Age | -0.05 | 0.02 | -0.09 – -0.02 | **0.003** |
| Gelling agent [Starch] x Age | -0.04 | 0.02 | -0.07 – -0.01 | **0.008** |
| Gelling agent [Blend] x Age | 0.00 | 0.02 | -0.04 – 0.05 | 0.916 |
| **Random Effects** | | | | |
| σ^2^ | 3.75 | | | |
| τ_00_ _Participant_ | 0.56 | | | |
| τ_00_ _Sample_ | 0.47 | | | |
| ICC | 0.22 | | | |
| N _Sample_ | 12 | | | |
| N _Participant_ | 147 | | | |
| Observations | 588 | | | |
| Marginal R^2^ / Conditional R^2^ | 0.120 / 0.311 | | | |

Note: Data was analyzed with a linear mixed model (estimated using REML and *nloptwrap* optimizer) to predict Overall liking with gelling agent, centered age and sex (formula: Overall liking ~ gelling agent + age + sex + age: gelling agent). The model included participant and samples as random effects (formula: ~1 | participant, ~1 | sample). The model’s intercept corresponds to gelling agent = gelatine, sex = female, and age treated as 0. Standardized parameters were obtained by fitting the models on a standardized version of the dataset. 95 % confidence intervals (CIs) and *p*-values were computed using Kenward–Roger degrees of freedom. ICC = Intraclass Correlation Coefficient; τ_00_ denotes the variance in intercepts, σ^2^ denotes the residual variance. Results for pectin and blend should be interpreted cautiously due to limited sample size.

## 4 Questions

**Check-all-that-apply**

- Please check all attributes that apply to the sample’s appearance.
  - Glossy
  - Matt
  - Neither matt nor glossy
- Please place the sample between your front teeth and bite down. Check all attributes that apply to the bite sensation.
  - Smooth bite
  - Medium bite
  - Tough bite
- Please chew the sample normally. Check all attributes that apply to the texture and taste of the sample.
  - Soft
  - Medium hard
  - Hard
  - Sticky
  - Chewy
  - Fruity
  - Sweet
  - Sour

**Consumer Study**

**Screening: Inclusion / Exclusion Criteria**

- How old are you?	(Number in Years)
- Which gender do you identify with?	(Male, Female, Diverse, Prefer not to say)
- Do you have type 1 or type 2 diabetes?	(Yes, No)
- Do you have any of the following food allergies or intolerances? Please tick all options that apply.	(Soy, Milk, Gluten, None of the above)
- Are you pregnant or breastfeeding?	(Yes, No)
- Which type of diet do you follow?	(Mixed diet, Gluten-free, Vegetarian, Vegan)
- If you selected vegetarian or vegan:
  Would you be willing to consume animal-derived ingredients (e.g. gelatine, beeswax) as part of this study?	(Yes, No)

**Consumption Behavior**

- How often do you eat sweets?
  - Daily
  - 2–3 times per week
  - Once a week
  - Once a month
  - Never
- How often do you eat fruit gummies?
  - Daily
  - 2–3 times per week
  - Once a week
  - Once a month
  - Never

During the sensory session, participants evaluated four different fruit gummy samples, each identified by a random three-digit code. For each sample, they rated how much they liked the product’s color, aroma, taste, sweetness, fruitiness, stickiness during chewing, hardness during chewing, and the product overall. They also assessed the perceived intensity of the product’s color, smell, taste, sweetness, fruitiness, stickiness, and hardness using Just-About-Right (JAR) scales**. Liking items were answered on 9-point scales anchored with “not at all” (1) and “very much” (9). JAR items used 5-point scales, anchored from “much too weak” to “much too strong”.** All questions were asked in German.

**Hedonic Evaluation**

- How much do you like the **color** of the product?
- How much do you like the **aroma** of the product?
- How much do you like the **taste** of the product?
- How much do you like the **sweetness** of the product?
- How much do you like the **fruitiness** of the product?
- How much do you like the **stickiness** of the product when chewing?
- How much do you like the **hardness** of the product when chewing?
- How much do you like the **product overall**?

**Just-About-Right Evaluation**

- How do you rate the intensity of the **color** of the product?
- How do you rate the intensity of the **aroma** of the product?
- How do you rate the intensity of the **taste** of the product?
- How do you rate the intensity of the **sweetness** of the product?
- How do you rate the intensity of the **fruitiness** of the product?
- How do you rate the intensity of the **stickiness** of the product?
- How do you rate the intensity of the **hardness** of the product?

## 5 References

Hothorn, T., Bretz, F., & Westfall, P. (2002). *CRAN: Contributed Packages.* https://doi.org/10.32614/CRAN.package.multcomp

Lenth, R. V. (2025). emmeans: Estimated Marginal Means, aka Least-Squares Means. Advance online publication. https://doi.org/10.32614/CRAN.package.emmeans

Makowski, D., Lüdecke, D., Patil, I., Thériault, R., Ben-Shachar, M. S., & Wiernik, B. M. (2021). *CRAN: Contributed Packages.* https://doi.org/10.32614/CRAN.package.report
